# Supplementary figures and images for: Rapid and cost-effective nutrient content analysis of cotton leaves using near-infrared spectroscopy (NIRS)
Source: PeerJ. 2021 Mar 11;9:e11042. doi: 10.7717/peerj.11042 (PMC7956002; doi:10.7717/peerj.11042)

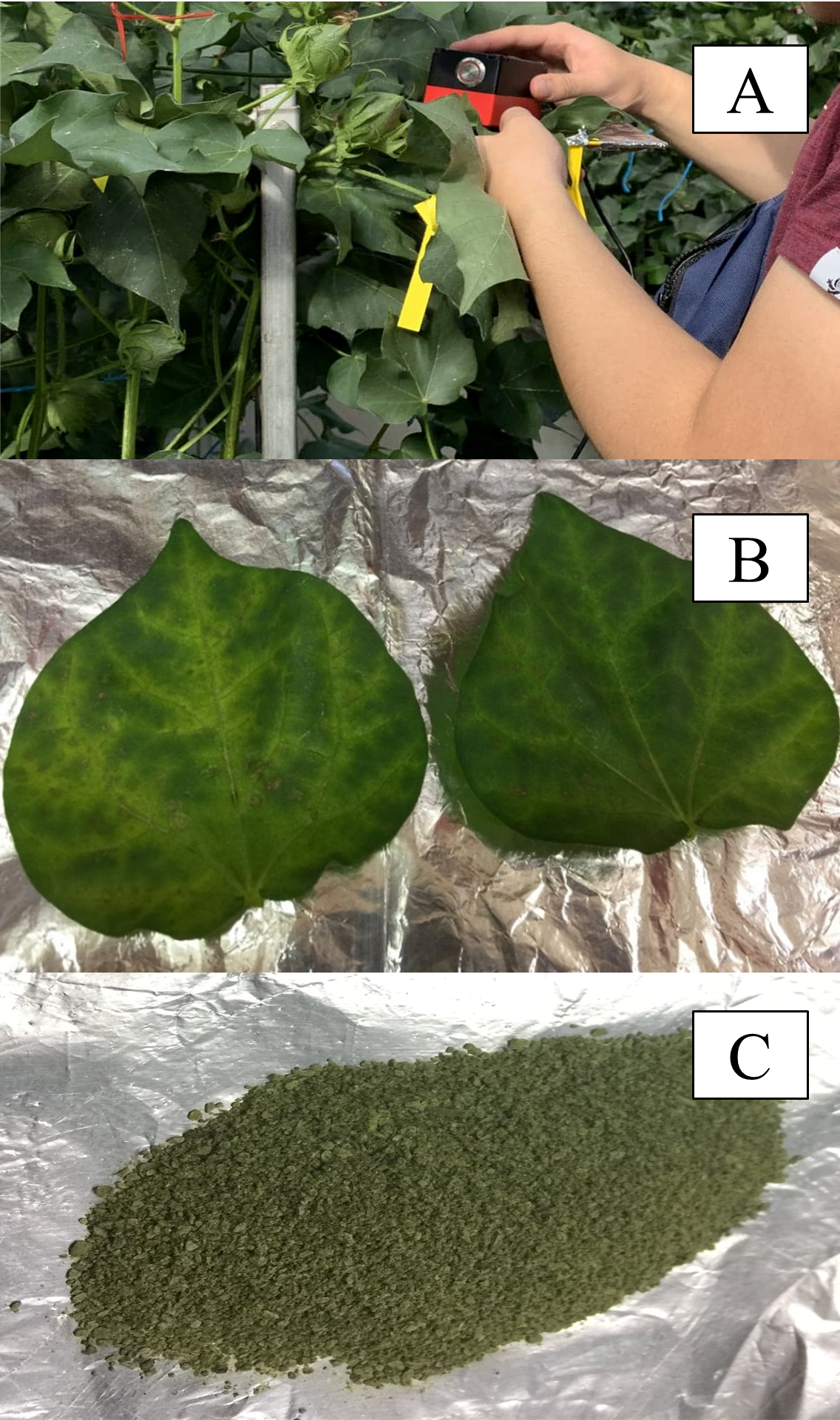

Supplement: Supplemental Information 2 — (A) Fresh and intact cotton leaves scanned using the PhoneLabTM spectrometer. (B) Fresh and removed cotton leaves 1 hour after removal from plant prepared for scanning on aluminium foil background. (C) Dried & ground cotton leaves sieved through a 0.25 mm sieve prepared for scanning on aluminium foil background. [file peerj-09-11042-s002.png]
