# Supplementary material for: Rapid and cost-effective nutrient content analysis of cotton leaves using near-infrared spectroscopy (NIRS)
Source: PeerJ. 2021 Mar 11;9:e11042. doi: 10.7717/peerj.11042 (PMC7956002; doi:10.7717/peerj.11042)
Supplement: Supplemental Information 3 — The statistival distribution summary is divided into macronutrient and micronutrient. The summary includes the minimum, 25th interval, mean, median, 75th interval, and maximum R2 value. [file peerj-09-11042-s003.docx]

|  | Macronutrient | | Micronutrient | |
| --- | --- | --- | --- | --- |
|  | **Calibration** | **Validation** | **Calibration** | **Validation** |
| Minimum | 0.89 | 0.76 | 0.76 | 0.36 |
| 25th interval | 0.90 | 0.77 | 0.82 | 0.64 |
| Median | 0.93 | 0.85 | 0.89 | 0.70 |
| Mean | 0.93 | 0.84 | 0.86 | 0.64 |
| 75th interval | 0.95 | 0.91 | 0.91 | 0.73 |
| Maximum | 0.97 | 0.94 | 0.92 | 0.76 |
| Interquartile range (IQR) | 0.05 | 0.14 | 0.09 | 0.09 |
