# Supplementary material for: Rapid and cost-effective nutrient content analysis of cotton leaves using near-infrared spectroscopy (NIRS)
Source: PeerJ. 2021 Mar 11;9:e11042. doi: 10.7717/peerj.11042 (PMC7956002; doi:10.7717/peerj.11042)
Supplement: Supplemental Information 4 — The accuracy parameters include the R2, Lin’s concordance, root mean square error (RMSE), and bias. Values are mean of 50 realisations of random data split (n = 375), 75:25 calibration: validation. [file peerj-09-11042-s004.docx]

| Nutrient | Validation | | | |
| --- | --- | --- | --- | --- |
|  | **R^2^** | **Concordance** | **RMSE (%)** | **Bias (%)** |
| Total Nitrogen | 0.94 | 0.96 | 0.20 | 0.00 |
| Phosphorus | 0.76 | 0.86 | 0.07 | 0.00 |
| Potassium | 0.76 | 0.85 | 0.33 | 0.01 |
| Calcium | 0.91 | 0.94 | 0.36 | 0.00 |
| Magnesium | 0.80 | 0.88 | 0.09 | 0.00 |
| Sulfur | 0.89 | 0.93 | 0.17 | -0.01 |
| Petiole Total N^*^ | 0.59 | 0.71 | 0.36 | 0.01 |
| Nitrate-N^+^ | 0.41 | 0.58 | 706.46 | -43.55 |

* n = 115,

^+^ RMSE (mg/kg), Bias (mg/kg)
