# Supplementary material for: Rapid and cost-effective nutrient content analysis of cotton leaves using near-infrared spectroscopy (NIRS)
Source: PeerJ. 2021 Mar 11;9:e11042. doi: 10.7717/peerj.11042 (PMC7956002; doi:10.7717/peerj.11042)
Supplement: Supplemental Information 5 — The accuracy parameters include the R2, Lin’s concordance, root mean square error (RMSE), and bias. Values are mean of 50 realisations of random data split (n = 375), 75:25 calibration: validation. [file peerj-09-11042-s005.docx]

| Nutrient | Validation | | | |
| --- | --- | --- | --- | --- |
|  | **R^2^** | **Concordance** | **RMSE (mg/kg)** | **Bias (mg/kg)** |
| Iron | 0.70 | 0.82 | 74.94 | -4.09 |
| Manganese | 0.75 | 0.85 | 39.74 | -1.11 |
| Copper | 0.65 | 0.79 | 1.15 | -0.04 |
| Zinc | 0.36 | 0.56 | 6.54 | -0.25 |
| Molybdenum | 0.73 | 0.84 | 299.95 | -12.16 |
| Boron | 0.64 | 0.79 | 18.09 | 0.15 |
| Chloride | 0.73 | 0.83 | 1900.00 | 0.00 |
| Sodium | 0.76 | 0.86 | 400.00 | 0.00 |
