# Supplementary material for: Rapid and cost-effective nutrient content analysis of cotton leaves using near-infrared spectroscopy (NIRS)
Source: PeerJ. 2021 Mar 11;9:e11042. doi: 10.7717/peerj.11042 (PMC7956002; doi:10.7717/peerj.11042)
Supplement: Supplemental Information 6 — The validation accuracy parameters includes the R2, Lin’s concordance, root mean square error (RMSE), and bias which are the mean of 50 realisations of random data split, 75:25 calibration: validation. [file peerj-09-11042-s006.docx]

| Macronutrient | Dried & Ground Validation | | | | Fresh & Removed Validation | | | |
| --- | --- | --- | --- | --- | --- | --- | --- | --- |
|  | **R^2^** | **Concordance** | **RMSE (%)** | **Bias (%)** | **R^2^** | **Concordance** | **RMSE** | **Bias** |
| Total Nitrogen | 0.92 | 0.94 | 0.21 | -0.01 | 0.86 | 0.91 | 0.29 | 0.00 |
| Phosphorus | 0.64 | 0.77 | 0.09 | 0.00 | 0.59 | 0.74 | 0.09 | 0.00 |
| Potassium | 0.76 | 0.85 | 0.37 | 0.00 | 0.76 | 0.85 | 0.37 | -0.02 |
| Calcium | 0.92 | 0.94 | 0.36 | -0.01 | 0.81 | 0.88 | 0.53 | 0.02 |
| Magnesium | 0.76 | 0.85 | 0.09 | 0.00 | 0.74 | 0.83 | 0.09 | 0.00 |
| Sulfur | 0.88 | 0.92 | 0.19 | -0.01 | 0.66 | 0.79 | 0.32 | -0.01 |
| Petiole Total N^*^ | 0.56 | 0.70 | 0.38 | 0.01 | 0.56 | 0.70 | 0.38 | -0.01 |
| Nitrate-N ^+^ | 0.36 | 0.53 | 685.52 | -7.39 | 0.25 | 0.44 | 713.09 | -69.20 |

* n = 115, ^+^ RMSE (mg/kg) & Bias (mg/kg)

| Micronutrient | Dried & Ground Validation | | | | Fresh & Removed Validation | | | |
| --- | --- | --- | --- | --- | --- | --- | --- | --- |
|  | **R^2^** | **Concordance** | **RMSE (%)** | **Bias (%)** | **R^2^** | **Concordance** | **RMSE** | **Bias** |
| Iron | 0.78 | 0.86 | 64.2 | -2.74 | 0.69 | 0.80 | 76.29 | -5.01 |
| Manganese | 0.76 | 0.85 | 44.14 | -1.89 | 0.73 | 0.83 | 45.59 | -0.80 |
| Copper | 0.65 | 0.78 | 1.10 | -0.03 | 0.50 | 0.68 | 1.35 | 0.03 |
| Zinc | 0.21 | 0.41 | 8.07 | -0.25 | 0.33 | 0.52 | 7.02 | -0.15 |
| Molybdenum | 0.70 | 0.81 | 203.98 | 0.76 | 0.54 | 0.71 | 255.47 | -7.97 |
| Boron | 0.65 | 0.79 | 19.36 | 0.90 | 0.71 | 0.82 | 17.44 | -0.61 |
| Chloride | 0.68 | 0.80 | 2300.00 | 0.00 | 0.60 | 0.74 | 2700.00 | 0.01 |
| Sodium | 0.58 | 0.72 | 400.00 | 0.00 | 0.26 | 0.42 | 500.00 | 0.00 |
