# Supplementary material for: Rapid and cost-effective nutrient content analysis of cotton leaves using near-infrared spectroscopy (NIRS)
Source: PeerJ. 2021 Mar 11;9:e11042. doi: 10.7717/peerj.11042 (PMC7956002; doi:10.7717/peerj.11042)
Supplement: Supplemental Information 7 — The validation accuracy parameters include the R2, Lin’s concordance, root mean square error (RMSE), and bias which are the mean of 50 realisations of random data split, 75:25 calibration: validation. [file peerj-09-11042-s007.docx]

| Macronutrient | Fresh & Removed Validation | | | | Fresh & Intact Validation | | | |
| --- | --- | --- | --- | --- | --- | --- | --- | --- |
|  | **R^2^** | **Concordance** | **RMSE (%)** | **Bias (%)** | **R^2^** | **Concordance** | **RMSE** | **Bias** |
| Total Nitrogen | 0.82 | 0.86 | 0.28 | -0.01 | 0.77 | 0.83 | 0.26 | 0.01 |
| Phosphorus | 0.19 | 0.34 | 0.11 | -0.01 | 0.12 | 0.28 | 0.11 | -0.00 |
| Potassium | 0.73 | 0.80 | 0.41 | -0.01 | 0.71 | 0.80 | 0.44 | -0.00 |
| Calcium | 0.36 | 0.52 | 0.56 | -0.03 | 0.51 | 0.67 | 0.63 | -0.05 |
| Magnesium | 0.62 | 0.73 | 0.12 | 0.00 | 0.61 | 0.73 | 0.11 | -0.01 |
| Sulfur | 0.21 | 0.30 | 0.38 | 0.04 | 0.26 | 0.46 | 0.38 | 0.00 |
| Petiole Total N | 0.13 | 0.15 | 0.31 | -0.01 | 0.11 | 0.20 | 0.34 | 0.00 |
| Nitrate-N^+^ | 0.17 | 0.15 | 772.09 | -106.41 | 0.15 | 0.23 | 832.85 | -33.06 |

^+^ RMSE (mg/kg), Bias (mg/kg)

| Micronutrient | Fresh & Removed Validation | | | | Fresh & Intact Validation | | | |
| --- | --- | --- | --- | --- | --- | --- | --- | --- |
|  | **R^2^** | **Concordance** | **RMSE (%)** | **Bias (%)** | **R^2^** | **Concordance** | **RMSE** | **Bias** |
| Iron | 0.38 | 0.48 | 85.65 | -3.19 | 0.60 | 0.67 | 68.85 | -1.44 |
| Manganese | 0.13 | 0.30 | 60.62 | -3.29 | 0.28 | 0.45 | 52.94 | -1.16 |
| Copper | 0.07 | 0.19 | 1.15 | -0.08 | 0.08 | 0.24 | 1.26 | -0.13 |
| Zinc | 0.14 | 0.16 | 9.76 | -0.70 | 0.12 | 0.19 | 9.06 | -0.83 |
| Molybdenum | 0.12 | 0.19 | 176.38 | -23.83 | 0.12 | 0.27 | 166.22 | -14.88 |
| Boron | 0.73 | 0.80 | 20.08 | -0.65 | 0.74 | 0.81 | 18.95 | 0.11 |
| Chloride | 0.68 | 0.76 | 2800.00 | 0.01 | 0.71 | 0.79 | 2700.00 | -0.01 |
| Sodium | 0.58 | 0.71 | 300.00 | 0.00 | 0.60 | 0.73 | 300.00 | 0.00 |
