# Supplementary material for: Rapid and cost-effective nutrient content analysis of cotton leaves using near-infrared spectroscopy (NIRS)
Source: PeerJ. 2021 Mar 11;9:e11042. doi: 10.7717/peerj.11042 (PMC7956002; doi:10.7717/peerj.11042)
Supplement: Supplemental Information 8 — The matrix identifies deficient or excessive levels of various macro- and micronutrients in a 25 random cotton leave samples from the Australian Cotton Research Institute (ACRI). The elements were compiled into macronutrients (N, P, K, S, Ca, Mg) and micronutrients (Na, Zn, Fe, Cu, Mn, B). The classification error rates were calculated by subtracting the measured percentage by the predicted percentage. [file peerj-09-11042-s008.docx]

|  | | Macronutrients | | | | |
| --- | --- | --- | --- | --- | --- | --- |
|  |  | **Predicted nutrients** | | |  |  |
|  |  | **High** | **Low** | **Normal** | **Total (measured)** | **Error** |
| Measured | **High** | 40.7 | 0.0 | 4.7 | 45.3 | 4.7 |
|  | **Low** | 0.0 | 28.7 | 4.7 | 33.3 | 4.7 |
|  | **Normal** | 2.7 | 0.7 | 18.0 | 21.3 | 3.3 |
| Total Classification Error | | | | | | **12.7** |

|  | | Micronutrients | | | | |
| --- | --- | --- | --- | --- | --- | --- |
|  |  | **Predicted nutrients** | | |  |  |
|  |  | **High** | **Low** | **Normal** | **Total (measured)** | **Error** |
| Measured | **High** | 30.7 | 0.0 | 2.0 | 32.7 | 2.0 |
|  | **Low** | 0.0 | 38.0 | 4.0 | 42.0 | 4.0 |
|  | **Normal** | 0.7 | 6.7 | 18.0 | 25.3 | 7.3 |
| Total Classification Error | | | | | | **13.3** |
