# Supplementary material for: Rapid and cost-effective nutrient content analysis of cotton leaves using near-infrared spectroscopy (NIRS)
Source: PeerJ. 2021 Mar 11;9:e11042. doi: 10.7717/peerj.11042 (PMC7956002; doi:10.7717/peerj.11042)
Supplement: Supplemental Information 9 — The reasoning includes some prove that the cubist method has shown to be superior compared to PLSR in this study and other studies. [file peerj-09-11042-s009.docx]

**Cubist vs. Partial Least Square Regression (PLSR)**

We show in comparison that Cubist and PLSR methods are comparable, and in some cases are superior to PLSR. Thus, we use the method. The same pre-treatments were used for both calibration method. The validation R^2^ and RMSE were compared and can be seen in the table below.

| **Cubist** | | | **PLSR** | | |
| --- | --- | --- | --- | --- | --- |
| **Nutrient** | **R2** | **RMSE** | | **R2** | **RMSE** |
| Boron (mg/kg) | 0.74 | 15.21 | | 0.73 | 15.48 |
| Calcium (%) | 0.91 | 0.36 | | 0.90 | 0.38 |
| Chloride (%) | 0.72 | 0.19 | | 0.62 | 0.22 |
| Copper (mg/kg) | 0.67 | 1.12 | | 0.68 | 1.09 |
| Iron (mg/kg) | 0.73 | 73.52 | | 0.70 | 76.40 |
| Magnesium (%) | 0.83 | 0.08 | | 0.80 | 0.09 |
| Manganese (mg/kg) | 0.78 | 37.04 | | 0.81 | 34.28 |
| Molybdenum (mg/kg) | 0.78 | 260.05 | | 0.74 | 281.51 |
| Phosphorus (mg/kg) | 0.78 | 0.07 | | 0.75 | 0.07 |
| Potassium (%) | 0.82 | 0.29 | | 0.74 | 0.34 |
| Sodium (%) | 0.79 | 0.04 | | 0.79 | 0.04 |
| Sulfur (%) | 0.89 | 0.17 | | 0.85 | 0.20 |
| Total Nitrogen (%) | 0.97 | 0.14 | | 0.95 | 0.18 |
| Zinc (mg/kg) | 0.36 | 6.55 | | 0.52 | 5.13 |
| Nitrate | 0.48 | 644.54 | | 0.53 | 617.91 |

The Cubist method has been widely used in Vis-NIR soil studies. Many studies have demonstrated the superiority of Cubist over PLSR, for example:

Peng, J., Biswas, A., Jiang, Q., Zhao, R., Hu, J., Hu, B., & Shi, Z. (2019). Estimating soil salinity from remote sensing and terrain data in southern Xinjiang Province, China. *Geoderma*, *337*, 1309-1319.

- Showed that Cubist (R2 = 0.91, RMSE = 5.18 dS m−1) performed better compared to PLSR (R2 = 0.66, RMSE = 10.46 dS m−1) in determining soil salinity

Minasny, B., & McBratney, A. B. (2008). Regression rules as a tool for predicting soil properties from infrared reflectance spectroscopy. *Chemometrics and intelligent laboratory systems*, *94*(1), 72-79.

- Showed that Cubsit models had lower RMSE than PLSR in predicting soil total C, CEC and clay content.

Minasny, B., McBratney, A. B., Stockmann, U., & Hong, S. Y. (2013). Cubist, a Regression Rule Approach for Use in Calibration of NIR Spectra. *Picking up Good Vibrations*, 630.
